# Survival from cancer of the stomach in England and Wales up to 2001

**DOI:** 10.1038/sj.bjc.6604575

**Published:** 2008-09-23

**Authors:** S Rao, D Cunningham

**Affiliations:** 1 Department of Medicine, Royal Marsden Hospital, Downs Road, Sutton, Surrey SM2 5PT, UK

Gastric cancer is characterised by nonspecific symptoms and thus patients often present at an advanced stage. Patients may present with weight loss, anorexia, epigastric discomfort, and more infrequently, early satiety or vomiting of blood. Diagnosis is by endoscopy and biopsy and CT scans of the chest, abdomen and pelvis are required to stage the disease. Endoscopic ultrasound (EUS) allows direct visualisation of the gastric mucosa and has an accuracy of up to 90% for T staging and 75% for N staging, which is superior to CT scan and was widely implemented towards the late 1990s (Dittler and Siewert, 1993; Matsumoto *et al*, 2000). Staging laparoscopy has become an accepted pretreatment evaluation for patients with localised disease for detecting occult metastatic disease not visible on CT scan. This probably had the greatest impact on staging in the 1990s when helical CT scans were not routinely employed and detection rates of occult metastases were up to 37% in some studies (Lowy *et al*, 1996; Asencio *et al*, 1997; Burke *et al*, 1997).

Surgical resection was the cornerstone of treatment for patients with localised gastric cancer during this period and many studies evaluating surgical techniques were conducted. Several studies demonstrated that perioperative mortality for gastrectomy was inversely related to the institutional gastrectomy volume (Birkmeyer *et al*, 2002; Hannan *et al*, 2002). This has led to the centralisation of care for gastric cancer with surgery generally performed at larger centres by specialist surgeons.

Adjuvant therapy was not routine practice at this time, as the supporting evidence was from relatively small studies. However, a large randomised prospective study of 503 patients by the Medical Research Council reported an improved progression-free survival for perioperative chemotherapy with epirubicin, cisplatin and 5FU (ECF) plus surgery *vs* surgery alone (HR 0.70 (95% CI: 0.56–0.88), *P*=0.002) (Allum *et al*, 2005). The long-term follow-up for this trial was reported and this has now translated into an overall survival (OS) benefit for the perioperative chemotherapy arm (HR 0.75 (95% CI: 0.60–0.93), *P*=0.009) (Cunningham *et al*, 2006). These results confirm the benefit of perioperative chemotherapy and demonstrated an acceptable postoperative mortality rate of 6%. Thus, perioperative chemotherapy plus surgery has been adopted as standard practice in the United Kingdom.

The management of advanced disease has developed significantly during the 1990s with a change in clinical practice. Several randomised studies demonstrated superior OS for chemotherapy compared with best supportive care (Murad *et al*, 1993; Glimelius *et al*, 1994). Trials were then conducted to evaluate combination chemotherapy and ECF showed superior efficacy and quality of life in two large randomised studies (Waters *et al*, 1999; Ross *et al*, 2002). Hence, ECF became the reference regimen for advanced gastric cancer in the latter part of the 1990s. The Cochrane Review of chemotherapy for advanced gastric cancer has recently concluded that among the current chemotherapy combinations, ECF demonstrated the best efficacy and tolerance (Wagner *et al*, 2005).

A national study of 1002 patients evaluated the substitution of capecitabine for infused-5FU and oxaliplatin for cisplatin in the original ECF regimen in patients with previously untreated advanced oesophago-gastric cancer. It reported that capecitabine and oxaliplatin were as effective as 5FU and cisplatin. Furthermore, OS was longer with epirubicin, oxaliplatin and capecitabine (EOX) than ECF (median OS of 11.4 months) with a hazard ratio for death for EOX of 0.80, 95% CI: 0.66–0.97; (*P*=0.02) (Cunningham *et al*, 2008).

There has been a consistent rise in 1-year survival of gastric cancer between 1986 and 1999, and this reflects decreased postoperative mortality and the use of chemotherapy for advanced disease. There was a small improvement in 5-year survival, which may be because of improved staging, patient selection for curative resection and decreased postoperative mortality.

The deprivation gap is most marked in men for short-term survival and has widened over time. One possible explanation is that the patients from the more deprived group were less likely to be referred to specialist centres for surgery until the late 1990s.

Predicted survival from period analysis suggests a continuing increase in short- and long-term survival. There has been considerable progress in the treatment of gastric cancer over the last 10 years. Staging of localised disease has improved with the use of EUS and laparoscopy, and more recently FDG PET has been employed to help identify metastatic deposits. Perioperative chemotherapy has now demonstrated a clear survival benefit for localised disease and this combined with the use of specialist surgical centres is likely to improve the outcome for these patients. Chemotherapy for advanced gastric cancer has produced a modest survival advantage and new chemotherapy combinations and targeted therapy may increase this further.

Since the late 1990s multidisciplinary teams have been established to ensure the optimal staging, diagnosis and management of gastric cancer. Cancers of the body and antrum of the stomach are associated with *Helicobacter pylori* infection and research is underway to evaluate the relationship of this infection with other possible aetiogical factors, such as tobacco and diet. Gene expression profiling and molecular markers are being studied to identify potential prognostic and predictive factors for gastric cancer.

## References

[bib1] Allum W, Cunningham D, Weeden S (2005) Perioperative chemotherapy in operable gastric and lower oesophageal cancer: A randomised, controlled trial (the MAGIC trial, ISRCTN 93793971). Proc Am Soc Clin Oncol. Abstract 998

[bib2] Asencio F, Aguilo J, Salvador JL, Villar A, De la ME, Ahamad M, Escrig J, Puche J, Viciano V, Sanmiguel G, Ruiz J (1997) Video-laparoscopic staging of gastric cancer. A prospective multicenter comparison with noninvasive techniques. Surg Endosc 11: 1153–1158 9373284 10.1007/s004649900559

[bib3] Birkmeyer JD, Siewers AE, Finlayson EV, Stukel TA, Lucas FL, Batista I, Welch HG, Wennberg DE (2002) Hospital volume and surgical mortality in the United States. N Engl J Med 346: 1128–1137 11948273 10.1056/NEJMsa012337

[bib4] Burke EC, Karpeh MS, Conlon KC, Brennan MF (1997) Laparoscopy in the management of gastric adenocarcinoma. Ann Surg 225: 262–267 9060581 10.1097/00000658-199703000-00004 PMC1190675

[bib5] Cunningham D, Allum WH, Stenning SP, Thompson JN, Van de Velde CJ, Nicolson M, Scarffe JH, Lofts FJ, Falk SJ, Iveson TJ, Smith DB, Langley RE, Verma M, Weeden S, Chua YJ, MAGIC Trial Participants (2006) Perioperative chemotherapy versus surgery alone for resectable gastroesophageal cancer. N Engl J Med 355: 11–20 16822992 10.1056/NEJMoa055531

[bib6] Cunningham D, Starling N, Rao S, Iveson T, Nicolson M, Coxon F, Middleton G, Daniel F, Oates J, Norman AR, Upper Gastrointestinal Clinical Studies Group of the National Cancer Research Institute of the United Kingdom (2008) Capecitabine and oxaliplatin for advanced esophago-gastric cancer. N Engl J Med 358: 36–46 18172173 10.1056/NEJMoa073149

[bib7] Dittler HJ, Siewert JR (1993) Role of endoscopic ultrasonography in gastric carcinoma. Endoscopy 25: 162–166 8491133 10.1055/s-2007-1010276

[bib8] Glimelius B, Hoffman K, Haglund U, Nyren O, Sjoden PO (1994) Initial or delayed chemotherapy with best supportive care in advanced gastric cancer. Ann Oncol 5: 189–190 8186165 10.1093/oxfordjournals.annonc.a058778

[bib9] Hannan EL, Radzyner M, Rubin D, Dougherty J, Brennan MF (2002) The influence of hospital and surgeon volume on in-hospital mortality for colectomy, gastrectomy, and lung lobectomy in patients with cancer. Surgery 131: 6–15 11812957 10.1067/msy.2002.120238

[bib10] Lowy AM, Mansfield PF, Leach SD, Ajani J (1996) Laparoscopic staging for gastric cancer. Surgery 119: 611–614 8650600 10.1016/s0039-6060(96)80184-x

[bib11] Matsumoto Y, Yanai H, Tokiyama H, Nishiaki M, Higaki S, Okita K (2000) Endoscopic ultrasonography for diagnosis of submucosal invasion in early gastric cancer. J Gastroenterol 35: 326–331 10832666 10.1007/s005350050356

[bib12] Murad AM, Santiago FF, Petroianu A, Rocha PR, Rodrigues MA, Rausch M (1993) Modified therapy with 5-fluorouracil, doxorubicin, and methotrexate in advanced gastric cancer. Cancer 72: 37–41 8508427 10.1002/1097-0142(19930701)72:1<37::aid-cncr2820720109>3.0.co;2-p

[bib13] Ross P, Nicolson M, Cunningham D, Valle J, Seymour M, Harper P, Price T, Anderson H, Iveson T, Hickish T, Lofts F, Norman A (2002) Prospective randomized trial comparing mitomycin, cisplatin, and protracted venous-infusion fluorouracil (PVI 5-FU) With epirubicin, cisplatin, and PVI 5-FU in advanced esophagogastric cancer. J Clin Oncol 20: 1996–2004 11956258 10.1200/JCO.2002.08.105

[bib14] Wagner AD, Grothe W, Behl S, Kleber G, Grothey A, Haerting J, Fleig WE (2005) Chemotherapy for advanced gastric cancer. Cochrane Database Syst Rev 2, doi:10.1002/14651858.CD004064.pub2 10.1002/14651858.CD004064.pub3 20238327

[bib15] Waters JS, Norman A, Cunningham D, Scarffe JH, Webb A, Harper P, Joffe JK, Mackean M, Mansi J, Leahy M, Hill A, Oates J, Rao S, Nicolson M, Hickish T (1999) Long-term survival after epirubicin, cisplatin and fluorouracil for gastric cancer: results of a randomized trial. Br J Cancer 80: 269–272 10390007 10.1038/sj.bjc.6690350 PMC2363002

